# Sensitive and Specific Target Sequences Selected from Retrotransposons of *Schistosoma japonicum* for the Diagnosis of Schistosomiasis

**DOI:** 10.1371/journal.pntd.0001579

**Published:** 2012-03-27

**Authors:** Jun-Jie Guo, Hua-Jun Zheng, Jing Xu, Xing-Quan Zhu, Sheng-Yue Wang, Chao-Ming Xia

**Affiliations:** 1 Department of Parasitology, Medical College of Soochow University, Suzhou, People's Republic of China; 2 Department of Parasitology, Qiqihaer Medical College, Qiqihaer, People's Republic of China; 3 Shanghai-MOST Key Laboratory of Health and Disease Genomics, Chinese National Human Genome Center at Shanghai, Shanghai, People's Republic of China; 4 State Key Laboratory of Veterinary Etiological Biology, Key Laboratory of Veterinary Parasitology of Gansu Province, Lanzhou Veterinary Research Institute, Chinese Academy of Agricultural Sciences, Lanzhou, People's Republic of China; National Institute of Parasitic Diseases China CDC, China

## Abstract

**Background:**

*Schistosomiasis japonica* is a serious debilitating and sometimes fatal disease. Accurate diagnostic tests play a key role in patient management and control of the disease. However, currently available diagnostic methods are not ideal, and the detection of the parasite DNA in blood samples has turned out to be one of the most promising tools for the diagnosis of schistosomiasis. In our previous investigations, a 230-bp sequence from the highly repetitive retrotransposon SjR2 was identified and it showed high sensitivity and specificity for detecting *Schistosoma japonicum* DNA in the sera of rabbit model and patients. Recently, 29 retrotransposons were found in *S. japonicum* genome by our group. The present study highlighted the key factors for selecting a new perspective sensitive target DNA sequence for the diagnosis of schistosomiasis, which can serve as example for other parasitic pathogens.

**Methodology/Principal Findings:**

In this study, we demonstrated that the key factors based on the bioinformatic analysis for selecting target sequence are the higher genome proportion, repetitive complete copies and partial copies, and active ESTs than the others in the chromosome genome. New primers based on 25 novel retrotransposons and SjR2 were designed and their sensitivity and specificity for detecting *S. japonicum* DNA were compared. The results showed that a new 303-bp sequence from non-long terminal repeat (LTR) retrotransposon (SjCHGCS19) had high sensitivity and specificity. The 303-bp target sequence was amplified from the sera of rabbit model at 3 d post-infection by nested-PCR and it became negative at 17 weeks post-treatment. Furthermore, the percentage sensitivity of the nested-PCR was 97.67% in 43 serum samples of *S. japonicum*-infected patients.

**Conclusions/Significance:**

Our findings highlighted the key factors based on the bioinformatic analysis for selecting target sequence from *S. japonicum* genome, which provide basis for establishing powerful molecular diagnostic techniques that can be used for monitoring early infection and therapy efficacy to support schistosomiasis control programs.

## Author Summary

Schistosomiasis remains a serious parasitic disease worldwide. Adequate case-finding is important and crucial for the effective control programs as schistosomiasis control programs are chiefly based on treatment of infected populations. However, the currently available diagnostic assays are not ideal, and DNA detection can provide useful alternative approaches for the sensitive and specific diagnosis of schistosomiasis, provided that reliable genetic markers are employed in the tests. However, different target sequences used for DNA detection of samples showed different sensitivity. In previous studies, we identified a 230-bp sequence of high sensitivity and specificity from the retrotransposon SjR2 of *Schistosoma japonicum*. Here, new primers based on 25 novel retrotransposons were designed and their sensitivities and specificities for detecting *S. japonicum* DNA were compared. Of these, a new 303-bp DNA sequence from the non-LTR retrotransposon (SjCHGCS19) had high sensitivity and specificity in detecting *S. japonicum* DNA. More importantly, we also found that both the SjCHGCS19 (303-bp sequence) and SjR2 (230-bp sequence) have high repetitive copies, higher genome proportions and active ESTs in the genome of *S. japonicum*. Our findings provide new insights into selecting suitable target sequences which may play a key role for the sensitive and specific detection of *S. japonicum* DNA.

## Introduction

Schistosomiasis is caused by *Schistosoma haematobium*, *S. mansoni*, *S. japonicum*, and less frequently, *S. mekongi* and *S. intercalatum*. It occurs in the tropics and subtropics and it is among the most important parasitic diseases worldwide, with a significant socio-economic impact [1]. The disease affects people living in 74 endemic countries, with approximately 120 million individuals being symptomatically infected and 20 million being severely affected [2], [3]. Moreover, schistosomiasis represents an increasing problem in non-endemic areas, due to the growing number of immigrants and tourists [4], [5]. As schistosomiasis control programs are chiefly based on treatment of infected populations, adequate case-finding is important for the effective consecution of the control programs. Herein, diagnosis plays a crucial role in the monitoring of early infection and therapy efficacy. However, the currently available diagnostic assays are not ideal, since the examination of eggs in stools, such as Kato-Katz assay, and detection of circulating antigens lack sensitivity due to low disease prevalence, post-treatment situations where chemotherapeutic agents mask the presence of existing disease and methodologically, antibody detection lacks specificity [6]. In addition, current ELISA methods cannot be used to evaluate the efficacy of chemical treatment as IgG antibody levels remained elevated despite the fact that the disease was cured, indicating that there may be false positive results [7].

In the last several years, several research groups have developed specific and sensitive PCR-based methods for detecting *S. japonicum*, *S. mansoni* and *S. haematobium* DNA from humans and the intermediate hosts [7]–[15]. These PCR assays have proven useful alternatives for the accurate diagnosis of human schistosomiasis. Numerous factors influence the sensitivity and specificity of PCR assays for the diagnosis of schistosomiasis, in particular, the target sequences selected for PCR amplification. Several research groups have developed PCR methods using various target sequences and these PCR assays showed different sensitivities for detection of *Schistosoma* genomic DNA. An 121-bp rDNA sequence was the major target sequence for detecting *S. mansoni* [8], [9], [11], [13]. Mitochondrial NADH I gene (*nad*1) has been used as genetic marker for detection of *S. mansoni* and *S. japonicum* DNA [15]–[17].

SjR2, a new RTE-like, non-long terminal repeat retrotransposon from *S. japonicum*, is firstly described by Laha et al. [18], and its 230-bp sequence was first used as target sequence in PCR and LAMP assays for detecting *S. japonicum* DNA [7], [12]. Recently, 29 retrotransposons were identified in the genome sequence of *S. japonicum* [19], including known Gulliver, SjR1, SjR2 and Sj-pido elements as well as 25 novel elements. In the present study, primers targeting these 25 novel retrotransposons were designed and used in PCR assays for detecting *S. japonicum* DNA, and their sensitivities and specificities were compared.

## Materials and Methods

### Ethics statement

This study was carried out in strict accordance with the recommendations in the Guide for the Care and Use of Laboratory Animals of the National Institutes of Health. The protocol was approved by the Committ-ee on the Ethics of Animal Experiments of the Soochow University (Permit Number: 2007–13).

All surgeries were performed under sodium pentobarbital anesthesia, and all efforts were made to mini-mize suffering of animals.

Serum samples of healthy individuals were obtained from Suzhou, Jiangsu Province, China. The protocol was approved by the Scientific Committee of the Soochow University. Serum samples of patients were obtained from the endemic area of Hunan Province, China. Written informed consent was obtained from all donors. Ethical clearance for the project was obtained from the Scientific Committee at the Hunan Institute of Parasitic Diseases, which is responsible for schistosomiasis control within the Hunan Province where targeted villages are located.

### Animal model of *S. japonicum* infection

Schistosome-infected snails were obtained from Jiangsu Institute of Parasitic Diseases, China. The institute provided live *Oncomelania hupensis* snails exposed to the Chinese strains of *S. japonicum*. All living snails were putting into a tray which was filled with 4/5 volume of water and exposed to a light source to induce shedding of live *S. japonicum* cercariae.

### Worms

*Clonochis sinensis* adult worms were provided by Prof. Kuiyang Zheng, Xuzhou Medical College, China, *Trichinella spiralis* adult worms were provided by Prof. Zhongquan Wang, Medical College of Zhengzhou University, China, and *S. mansoni* adult worms were obtained from Dr. Donato Cioli, Institute of Cell Biology, Monterotondo, Italy.

### Collection of samples in rabbit models

Six female New Zealand rabbits, weighing 1.8–2.2 kg, were randomly divided into three groups of two rabbits each. Blood collected before infection from all rabbits was served as negative control. Group I and Group II were percutaneously infected with 200 mixed sexual cercariae of *S. japonicum* (light infection), and Group III was percutaneously infected with 500 mixed sexual cercariae of *S. japonicum* (medium infection). On the seventh week, the eggs were detected in the feces. The EPG was 96 (Group I), 92 (Group II) and 397 (Group III), respectively. In Group I, between 1 and 7 weeks post-infection, blood were collected weekly. On the seventh week, the rabbits were anaesthetized with sodium pentobarbital and the adult worms and liver samples were collected. Between 1 and 24 weeks post-infection, blood was collected from rabbits in Group II. Serum of each rabbit was separated by centrifugation (1500 *g* for 15 min) and kept at −80°C until use.

### Treatment of infected rabbits and collection of samples

The rabbits infected with *S. japonicum* in Group III were treated with two doses of 150 mg/kg praziquantel on the seventh and eighth week post-infection. In this group, blood was collected weekly between 1 and 30 weeks post-infection (23 weeks post-treatment) and all of the rabbits were sacrificed on the last week, and no adult worms were found in portal system. Serum of each rabbits was separated by centrifugation (1500 *g* for 15 min) and kept at −80°C until use.

### Collection of human serum samples

Forty-three *S. japonicum*-positive serum samples of patients with positive Kato-Katz stool examination results were obtained from the endemic areas in Hunan Province, China, and the egg counts of these patients ranged from 8 to 1160 eggs per gram (EPG). 51 serum samples from healthy individuals were collected from healthy donors at the Center of Health Examination, the First Affiliated Hospital of Soochow University, Suzhou, Jiangsu Province, China, and were used as negative control to evaluate the specificity of the PCR assay.

### Extraction of DNA from liver homogenate of infected rabbits, adult *S. japonicum*, *S. mansoni*, *C. sinensis* and *T. spiralis*, and serum samples of infected rabbits and human serum samples

DNA from all of the collected samples was extracted using the method described by Steiner et al [20]. as modified by Xia et al. [7]. Briefly, 0.2 g liver homogenate samples was mixed with 200 µl distilled water, grinded and dissolved in 200 µl Triton-X-100 (10 mMol/L Tris-HCL; 0.45% Triton-X-100; 0.45% NP-40; 300 µg/mL protease K, pH 7.4) and were incubated at 60°C for 2 h with vigorous agitation for 10 min on a shaker, and then centrifuged at 12,000 *g* for 10 min. 300 µl supernatant of the liver homogenate was collected in a separate tube and incubated at 100°C for 10 min, centrifuged at 12,000 *g* for 10 min and the supernatant was collected.

DNA from adult worms was also extracted using the method modified by Xia et al [7]. Five adult worms of each *S. japonicum*, *S. mansoni*, *C. sinensis* and *T. spiralis* were homogenized in 300 µl physiological saline and then digested with equal volume of extraction buffer containing 3 mg/ml proteinase K, 0.1 mol/L Tris-HCl, pH 8.5, 0.05 mol/L EDTA, and 1% SDS. Then the mixture was incubated at 60°C for 1 h.

200 µl serum of infected rabbits or humans were diluted in 400 µl serum extraction buffer containing 150 mol/L NaCl, 10 mol/L EDTA, 10 mol/L Tris-HCl, pH 7.6, 2% SDS, 5 µg/ml salmon sperm DNA, 4 µg 25 mg/ml proteinase K, and were incubated at 37°C overnight.

All of the extraction mixtures (liver homogenate, adult samples and serum) were extracted twice with phenol, chloroform, isoamyl alcohol (25∶24∶1), once with chloroform, isoamyl alcohol (24∶1), and then precipitated with dehydrated alcohol and 3 M sodium acetate. The supernatant was discarded and the pellet was washed twice with 1 mL of 75% ethanol, finally the pellet was left to dry at 37°C for 30 min and then re-suspended with 100 µl of TE (10 mmol/L Tris-HCl, 1 mmol/L EDTA, pH 8.0) for DNA of liver homogenates and adult worms, and 20 µl of TE (10 mM Tris-HCl, 1 mM EDTA, pH 8.0) for DNA of serum samples, respectively.

### Polymerase chain reaction (PCR)

The DNA extracted from adult worms, liver and serum were used as the template. Primers were designed targeting the 25 novel retrotransposons repeat DNA sequences of *S. japonicum* (Table S1). PCR reaction (25 µl) contained 2.5 µl of buffer, 1.5 µl of 25 mmol/L MgCl_2_, 2 µl of 2.5 mmol/L dNTP, 0.5 µl of each 20 pmol/L primer, 0.4 µl of 5 U/L *Taq* polymerase (Takara) and 4 µl of template. The conditions for PCR were as follows (with the exception of SjCHGCS4 and SjCHGCS6 using 2-step PCR: 94°C for 4 min, followed by 30 cycles of 94°C for 30 s, 68°C for 60 s, and a final extension of 72°C for 7 min): 94°C for 3 min, followed by 35 cycles of 94°C for 60 s, Tm/(list in Table S1) for 60 s, 72°C for 60 s and a final extension of 72°C for 7 min. The PCR was performed using GeneAmp PCR System (Eppendorf, Hamburg, Germany). Finally, a 5 µl aliquot of the PCR product was run on a 2.0% agarose gel along with DNA ladder marker (Takara, Dalian) in TBE buffer containing 0.5 µg/ml ethidium bromide, and the bands were visualized under UV light on a transilluminator.

### Nested-PCR reaction

The nested-PCR reaction system was similar to the normal PCR as described above. The first-round amplification was carried out in the same manner except that the degenerate temperature was 55°C. Primers were designed on the basis of the SjCHGCS19 retrotransposons repreat DNA sequence of *S. japonicum*, the primers employed were P3 (5′-CCAAATCGCAACACTACGC-3′ (forward) and P4 (5′-ATCGGATTCTCCTTGTTCAT-3′) (reverse). DNA samples extracted from serum of rabbits and humans were used as the template. The expected length of the amplification product was 607 bp. Second-round amplification (nested-PCR) was carried out in the same manner as the first-round except that the DNA sample was a 1∶10000 dilution of the first-round PCR product and the degenerate temperature was 65°C. The sequences of these primers are listed in Table S1. The expected length of the amplification product was 303 bp.

### Plasmid of SjCHGCS19 clone

The first-round PCR product targeting SjCHGCS19 was cloned into plasmid by means of a pMD20-T II cloning reagent Kit (Tiangen, Beijing, China). Plasmid purification was done with a TIAN pure Mini Plasmid Kit (Tiangen, Beijing, China). Plasmids were quantified by spectrophotometry. Sequencing of the cloned amplification product confirmed that it was identical to part of the *S. japonicum* retrotransposon SjCHGCS19. The standard plasmid was tested in 10-dilution series by nested-PCR.

## Results

### Sensitivity and specificity of target sequences from 25 novel retrotransposons in nested-PCR assay

Primers were designed from the 25 novel retrotransposons of *S. japonicum* and a series of diluted genomic DNA of *S. japonicum* adults were used as the template. The target fragments were amplified by nested-PCR assay and the minimum amounts detectable were different among 25 new retrotransposons and SjR2. In addition to the 230-bp fragment from SjR2, a new 303-bp fragment from non-LTR retrotransposon (SjCHGCS19) displayed high sensitivity and specificity. Bioinformatic analysis showed that both the SjCHGCS19 (303-bp sequence) and SjR2 (230-bp sequence) have higher genome proportions (4.09% and 4.43%), higher repetitive complete copies (793 and 400) and partial copies (17,373 and 23,755), and higher EST numbers (39 and 79) than the other retrotranposons in the genome of *S. japonicum* (Table 1).

**Table 1:** Mobile-element character and the sensitivity and specificity of 26 *S.japonicum* retrotransposons by nested-PCR.

*LTR Retrotransposons*										
Retrotransposon	Proposed Group	Total Length (bp)	Genome Portion (%)	Complete Copies	Partial Copies	Activity ( ESTs, E<1E-50)	PurposeFragment Size(bp)	Sensitivity of PCR(2.15×10^n^ fg/µl)	Cross-reaction with *C.sinesis*	Cross-reaction with *S.mansoni*
***SjCHGCS1***	fugitive	5,249	1.45	188	5,208	24	1006	8	no	no
***SjCHGCS2***	Boudicca/Saci-3	4,945	0.49	40	2,062	25	968	7	yes	yes
***SjCHGCS3***	fugitive	4,451	0.31	28	1,679	12	949	8	no	no
***SjCHGCS4***	Saci5	4,710	0.04	10	130	8	725	8	no	no
***SjCHGCS5***	Saci5	2,148	0.19	79	1,184	5	697	7	yes	yes
***SjCHGCS6***	fugitive	4,839	0.28	18	2,085	9	691	9	no	no
***SjCHGCS7***	unknown	4,000	0.16	10	1,064	2	754	9	no	no
***SjCHGCS8***	BEL	4,865	0.08	3	401	1	679	11	no	yes
***SjCHGCS9***	Saci5	4,779	0.06	13	204	25	803	9	no	yes
***SjCHGCS10***	Boudicca/Saci-3	5,025	0.61	126	1,210	63	828	7	no	no
***SjCHGCS11***	Boudicca/Saci-3	6,488	0.21	4	987	31	664	8	no	yes
***SjCHGCS12***	fugitive	4,965	0.38	20	2,236	21	846	8	no	no
***SjCHGCS13***	fugitive	5,099	0.16	16	856	26	820	8	yes	no
***SjCHGCS14***	fugitive	5,211	0.08	6	294	29	721	7	yes	yes
***SjCHGCS15***	fugitive	4,711	1.10	15	8,707	11	681	8	no	yes
***SjCHGCS16***	BEL	7,232	0.18	4	737	15	352	8	no	yes
***SjCHGCS17***	BEL	6,960	1.10	4	389	16	698	8	no	no
***SjCHGCS18***	BEL	5,985	1.10	7	471	0	688	9	no	no
*Non-LTR Retrotransposons*										
***SjR2***	SR2	3,921	4.43	400	23,755	79	231	7	no	no
***SjCHGCS19***	SR2	3,145	4.09	793	17,373	39	607	6	no	yes
***SjCHGCS20***	SR2	3,578	2.56	223	14,801	26	859	8	yes	yes
***SjCHGCS21***	Perere-9	4,275	0.01	1	26	0	864	9	no	yes
***SjCHGCS22***	unknown	3,768	0.32	10	2,965	0	800	9	yes	no
*Penelope-like elements*										
***Sj-penelope1***	Cercyon	3,750	0.36	3	2,717	3	864	8	no	no
***Sj-penelope2***	Cercyon	2,734	0.40	34	2,989	8	883	8	yes	yes
***Sj-penelope3***	Cercyon	2,512	0.23	11	1,637	0	759	8	yes	no

Furthermore, to determine the limit of the 303-bp DNA fragment for detecting *S. japonicum* DNA, 10-fold serial dilutions of the standard plasmid clones of SjCHGCS19 and SjR2 were amplified by nested-PCR assay. The minimum detectable amount of standard plasmid from SjCHGCS19 was only 2.02 copies, whereas the 230-bp fragment from SjR2 was 10.2 copies.

**Figure 1:**
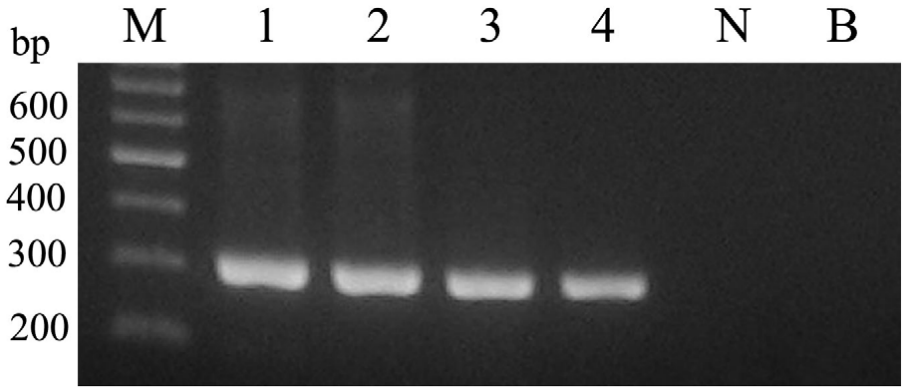
Detection of *Schistosoma japonicum* DNA by nested-PCR. DNA from adult *S. japonicum*, liver homogenate and serum were used as template. M represents a DNA size marker. Lanes 1–4 represent adult females of *S. japonicum*, adult males of *S. japonicum*, liver homogenate and serum of rabbit at 7 weeks post-infection, respectively. Lane N represents serum of non-infected rabbit (negative control). Lane B represents no template (blank) control.

**Figure 2:**
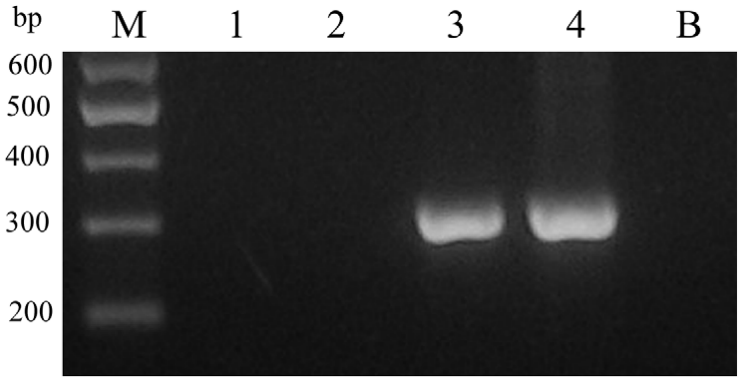
Specificity of nested-PCR for the detection of *Schistosoma japonicum* DNA. M represents a DNA size marker. Lanes 1–4 represent adult *Trichinella spiralis*, adult *Clonochis sinensis*, adult male *S. mansoni* and adult male *S. japonicum*, respectively. Lane B represents no template (blank) control.

In addition, the 303-bp DNA fragment was amplified by nested-PCR assay from male and female adults of *S. japonicum*, from liver homogenate and sera of infected rabbits (Figure 1). Interestingly, the target DNA was amplifiable from both *S. japonicum* and *S. mansoni*, but no cross-reaction was detected with DNA samples representing *C. sinensis* and *T. spirals* (Figure 2).

### Evaluation of the 303-bp target sequence for detection of early infection with *S. japonicum* and chemotherapy evaluation

**Figure 3:**
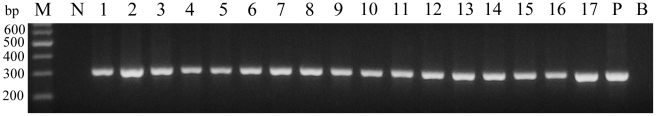
Early detection of *Schistosoma japonicum* DNA in rabbit serum samples. M represents a DNA size marker. Lane N represents serum of non-infected rabbit (negative control). Lanes 1–17 represent sera of rabbits at 3 d, 1–7 weeks, 9, 11, 13, 15, 17, 19, 20, 22, 24 w post-infection with 200 *S. japonicum* cercariae. Lane P represents adult *S. japonicum* (positive control). Lane B: represents no template (blank) control.

**Figure 4:**
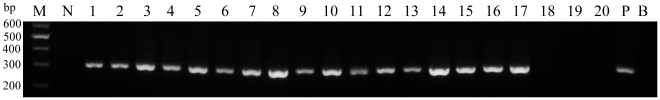
Evaluation of efficacy of chemotherapy. M represents a DNA size marker. Lane N represents serum of non-infected rabbit (negative control). Lanes 1–20 represent sera of 8–27 weeks post-infection with 500 *S. japonicum* cercariae. Lane P represents adult *S. japonicum* (positive control). Lane B represents no template (blank) control. *Rabbits were treated with praziquantel on the seventh and eighth week and all of the rabbits were sacrificed on the last week, and no adult worms were found in portal system. ** Rabbit sera at 1–7 weeks post-infection in group III showed coincident positive results.

To clarify the potential diagnostic value of the 303-bp DNA fragment for early infection and chemotherapy evaluation in the rabbit model, a series of rabbit sera in Group II (light infection) and Group III (praziquantel treatment) were examined by nested-PCR assay. As shown in Figure 3, the 303-bp DNA fragment was amplified at 3 d sera post-infection in Group II and could be amplified until 24 weeks post-infection by nested-PCR assay. The 303-bp DNA fragment was amplifiable between 1 and 24 weeks post-infection and it became negative from 25 weeks post-infection (17 weeks after praziquantel treatment) in Group III (Figure 4).

To illuminate the clinical utility of the 303-bp DNA from SjCHGCS19 as the target sequence for the diagnosis of human schistomomiasis, 43 human serum samples from patients with *S. japonicum* infection confirmed by stool examination and 51 serum samples from normal healthy individuals were examined. Of the 43 patient serum samples, 42 (97.67%) were positive by the nested-PCR assay. Of the 51 serum samples from healthy individuals, 49 (96.07%) were detected as negative by the nested-PCR assay. the percentages of sensitivity, specificity, positive predictive value (PPV) and negative predictive value (NPV) for nested-PCR assay were 97.67%, 96.07%, 97.67% and 96.07%%, respectively. For patients with heavy infection with *S. japonicum* (EPGs≥400) and medium infection (100≤EPG<400), the percentage of sensitivity was 100%. For patients with light infection (EPG<100), the percentage of sensitivity was 96.87% (Table 2, Figure S1).

**Table 2:** Detection of *S. japonicum* DNA in sera of patients with different EPGs by nested-PCR.

EPG	Positive	Negative	Positive rate (%)
400∼	2	0	100
100∼400	8	0	100
0∼100	32	1	96.87
total	42	1	97.67

## Discussion

Because schistosomiasis control programs are chiefly based on treatment of infected populations, adequate case-finding is important for the effective consecution of the control programs. Herein, accurate diagnosis plays a crucial role in the monitoring of early infection and therapy evaluation. Nucleic acid-based diagnosis has been used in the clinical testing of a wide variety of pathogenic infections, such as human immunodeficiency virus [21], *Mycobacterium tuberculosis* [22], *Plasmodium falciparum* [23], *Trypanosoma cruzi* [24] and *Leishmania braziliensis* [25]. In parasitic diseases such as schistosomiasis, it was possible to detect cell-free parasite DNA circulating in plasma, and this could be used to diagnose schistosomiasis. Importantly, nucleic acid-based diagnostic methods have the same diagnostic value as parasitological diagnostic methods. Recently, several PCR-based methods for detecting *Schistosoma* DNA from various samples have been developed [7]–[15]. Numerous factors may influence the sensitivity and specificity of PCR assays for the diagnosis of schistosomiasis, in particular the target sequences selected.

A 121-bp tandem repeat rDNA sequence was the major target sequence for detecting *S. mansoni* DNA in mouse serum samples and in human plasma by PCR [11], [13]. Although a real-time PCR assay using *nad*1 as target sequence for detecting *S. japonicum* DNA has proven highly sensitive, even for samples containing less than 10 EPG, it was negative for examining serum and urine samples from the infected pigs [14].

While previous efforts of PCR diagnosis of *S. mansoni* and *S. japonicum* infections have been relied on mitochondrial or rDNA sequences, we have focused on the sequence of the highly repetitive retrotransposon SjR2 in *S. japonicum*. In our previous studies, the 230-bp sequence from the highly repetitive retrotransposon SjR2 of *S. japonicum* was used as target sequence and it showed high sensitivity and specificity in detecting *S. japonicum* DNA, and the 230-bp fragment was amplified with DNA equivalent of 1.1 egg from feces [7]. In particular, the 230-bp sequence was able to be amplified from the sera of the rabbit model at 1 week post-infection, which is one week earlier than that of the 121-bp sequence in the mouse-*S. mansoni* model [7], [11], [12].

SjR2 was 3.9 kb in length and was constituted of a single open reading frame encoding a polyprotein with apurinic/apyrimidinic endonuclease and reverse transcriptase domains [18]. Phylogenetic analyses based on conserved domains of reverse transcriptase or endonuclease revealed that SjR2 belonged to the RTE clade of non-long terminal repeat retrotransposons and SR2 elements are members of a non-LTR retrotransposons typified by the RTE-1 non-LTR retrotransposon of *Caenorhabditis elegans* [26]. According to the phylogenetic tree, both SjCHGCS19 and SjR2 belonged to RTE clade of non-LTR retrontransposon (Figure S2). SjCHGCS19 showed the closest relationship with Perere-3 of *S. mansoni*, while SjR2 located in the same branch with SR2. At amino acids level, SjCHGCS19 showed 74% identity with Perere-3, while only 30% identity with SR2 and SjR2. Between SR2 and SjR2, the polyprotein showed 55% identity. Furthermore, hybridization analyses indicated that 10,000 copies of SjR2 were dispersed throughout the *S. japonicum* genome, accounting for up to 14% of the nuclear genome [18]. In our previous study, 29 retrotransposons were identified, including the known Gulliver, SjR1, SjR2 and Sj-pido elements as well as 25 novel elements, together constituting 19.8% of the genome. Of the 25 novel retrotransposons, 18 were LTR forms, four were non-LTR forms and three were Penelope-like elements—enigmatic retroelements that retain introns. Each type of retrotransposons was represented by 1 to 793 intact copies or hundreds to thousands of partial copies [19]. The non-LTR retrotransposons such as SjR1, SjR2, Sj-pido, SjCHGCS19, SjCHGCS20, SjCHGCS21 and SjCHGCS22 have significantly higher copy numbers, constituting 12.6% of the genome. We then wanted to determine whether there are any associations between the numbers of copies of target sequences and the sensitivity of the PCR detection.

In the present study, primers were designed to amplify the 25 novel *S. japonicum* retrotransposons. The target fragments were amplified by nested-PCR assay for comparing sensitivity and specificity of detecting *S. japonicum* DNA of the target sequences. The results showed that a new 303-bp fragment from the highly repetitive retrotransposon, SjCHGCS19, had high sensitivity for detecting *S. japonicum* DNA in addition to the 230-bp fragment from SjR2. Importantly, bioinformatic analysis of 26 *S. japonicum* retrotransposons showed that both the SjCHGCS19 (303-bp sequence) and SjR2 (230-bp sequence) have higher genome proportions, repetitive complete copies and partial copies, and active ESTs than the others in the chromosome genome (Table 1).

The minimum amount of the standard plasmid detectable using nested-PCR assay was 2.02 copies per reaction using the 303-bp target sequence from SjCHGCS19, whereas 10.2 copies per reaction of *S. japonicum* DNA was detected targeting the 230-bp sequence from SjR2 by the nested-PCR assay. This indicated that the 303-bp fragment from SjCHGCS19, as target sequence for detecting *S. japonicum* DNA, was more sensitive than the 230-bp fragment from SjR2 previously identified.

Additionally, as shown in Figure 1, we evaluated the specificity of the 303-bp target sequence. The expected product was amplified by the nested-PCR assay from *S. japonicum* male and female adults, from liver homogenate and from sera of infected rabbits. We did not find any false positive bands in non-infected rabbit sera. Interestingly, the target DNA was amplified from both *S. japonicum* and *S. mansoni*, but no cross-reaction was detected in DNA samples representing *C. sinensis* and *T. spirals* (Figure 2).

Furthermore, in the present study, the 303-bp sequence was amplified in rabbit sera at 3 d day post-infection, which is 4 d earlier than that of the 230-bp sequence in the rabbit-*S. japonicum* model by nested-PCR (Figure 3). The results showed that higher sensitivity is achieved using the 303-bp sequence for the detection of *S. japonicum* DNA in serum samples. In addition to the obvious advantage of an early diagnosis, our findings showed that the 303-bp target sequence might be valuable for the evaluation of chemotherapy efficacy, and it became negative at 25^th^ week post-infection (17 weeks after praziquantel treatment) by nested-PCR, and was 7 weeks longer than detection by using the 230-bp sequence from SjR2, indicating a higher sensitivity of the 303-bp sequence than that of the 230-bp sequence (Figure 4).

The effectiveness of the 303-bp target sequence was validated by examining serum samples from patients infected with *S. japonicum* (Figure S1). The findings showed that the sensitivity was 97.67%, and the specificity was 96.07% (Table 2). In particular, the PPV was 100% in patients with heavy infection (EPGs≧400) and medium infection (100≦EPG<400), indicating its high sensitivity. For patients with light infection (EPG<100), the percentage of sensitivity was 96.87%. Only 1 serum sample was detected as false negative, and this sample was from a patient with particularly low infection (EPG = 56). This could be due to little *S. japonicum* DNA in human blood circulation and/or DNA loss during template extraction procedures. There appeared to be a correlation between PCR results and the EPGs of the patients (Table 2). However, in this study, the examined number of patient serum samples was small. It is imperative to carry out further investigation using a large number of patient serum samples.

Since the prevalence of human infection with *S. japonicum* has been decreasing year by year [27], the rapid and reliable diagnosis of *Schistosoma* infection is central to the control as well as to the environmental monitoring and disease surveillance, especially for evaluation of treatment efficacy. DNA amplification assays provide alternative approaches for sensitive and specific diagnosis of *Schistosoma* infection, provided that reliable genetic markers are employed in the tests. Our results demonstrated that this new 303-bp sequence from non-LTR retrotransposon (SjCHGCS19) had high sensitivity and specificity for the detection of *S. japonicum* DNA, which may provide the new target sequence useful for the early diagnosis and for the evaluation of chemotherapy efficacy of schistosomiasis. More importantly, although many factors may affect the sensitivity and specificity in the target sequence selection, such as the length of target sequence, the specificity of the conserved sequence, the present study highlighted the key factors based on bioinformatic analysis for selecting a new perspective sensitive target sequence from genome sequences, which provides new insights into selecting suitable target sequence which may play a key role for the sensitive and specific detection of Schistosoma DNA. These findings would provide basis for establishing powerful molecular diagnostic techniques that can be used in clinical settings and as laboratory tools for surveillance and for environmental monitoring to support schistosomiasis control programs.

## Supporting Information

Figure S1 **Detection of** ***Schistosoma japonicum*** **DNA in patients with different intensities of infection.** M represents a DNA size marker. Lanes 1 and 2 represent serum samples from heavy-infected patients (EPG≧400). Lanes 3 and 4 represent serum samples from medium-infected patients (100≦EPGs<400). Lanes 5 and 6 represent serum samples from light-infected patients (EPGs<100). Lane N represents a serum sample from non-infected human (negative control). Lane P represents adult *S. japonicum* (positive control). Lane B represents no template (blank) control.
(TIF)

Figure S2 **Phylogenetic relationshiops of the reverse transcriptase domain of SjCHGCS19 with other non-LTR retrontransposons.** The tree was constructed by the Neighbor-Joining method. Numbers represent the percentage of replicate trees in which the associated taxa clustered together in the bootstrap test (1000 replicates). Rerere (DAA04497.1), Perere-3 (CAJ00236.1), Perere-9 (CAJ00246.1), SR1 (AAC06263.1) and SR2 (AAC24982.2) represent non-LTR retrotransposons of *Schistosoma mansoni*. SjR1 (AAC62955.1), Sj-pido (AY034003.1), SjR2 (AAK14815.1) and SjCHGCS19 (CAX83710.1) represent non-LTR retrotransposon of *S. japonicum*. CR1 (AAC60281.1) represents non-LTR retrotransposon of *Gallus gallus*, R2 (AAB59214.1) represents non-LTR retrotransposon of *Bombyx mori*, and R4 (AAA97394.1) represents non-LTR retrotransposon of *Ascaris lumbricoides*.
(DOCX)

Table S1 **The primers for the amplification of 26** ***Schistosoma japonicum*** **retrotransposons by PCR.**
(DOC)

Table S2 **STARD checklist for reporting of studies of diagnostic accuracy.**
(DOC)
