# Supplementary figures and images for: Sensitive and Specific Target Sequences Selected from Retrotransposons of Schistosoma japonicum for the Diagnosis of Schistosomiasis
Source: PLoS Negl Trop Dis. 2012 Mar 27;6(3):e1579. doi: 10.1371/journal.pntd.0001579 (PMC3313927; doi:10.1371/journal.pntd.0001579)

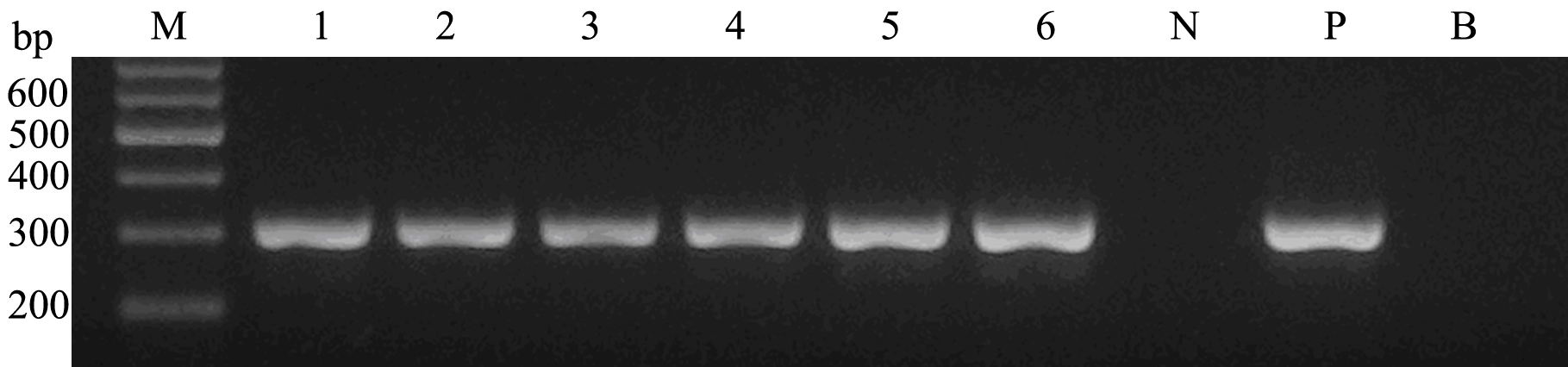

Supplement: Figure S1 — Detection of Schistosoma japonicum DNA in patients with different intensities of infection. M represents a DNA size marker. Lanes 1 and 2 represent serum samples from heavy-infected patients (EPG≧400). Lanes 3 and 4 represent serum samples from medium-infected patients (100≦EPGs<400). Lanes 5 and 6 represent serum samples from light-infected patients (EPGs<100). Lane N represents a serum sample from non-infected human (negative control). Lane P represents adult S. japonicum (positive control). Lane B represents no template (blank) control. (TIF) [file pntd.0001579.s001.tif]

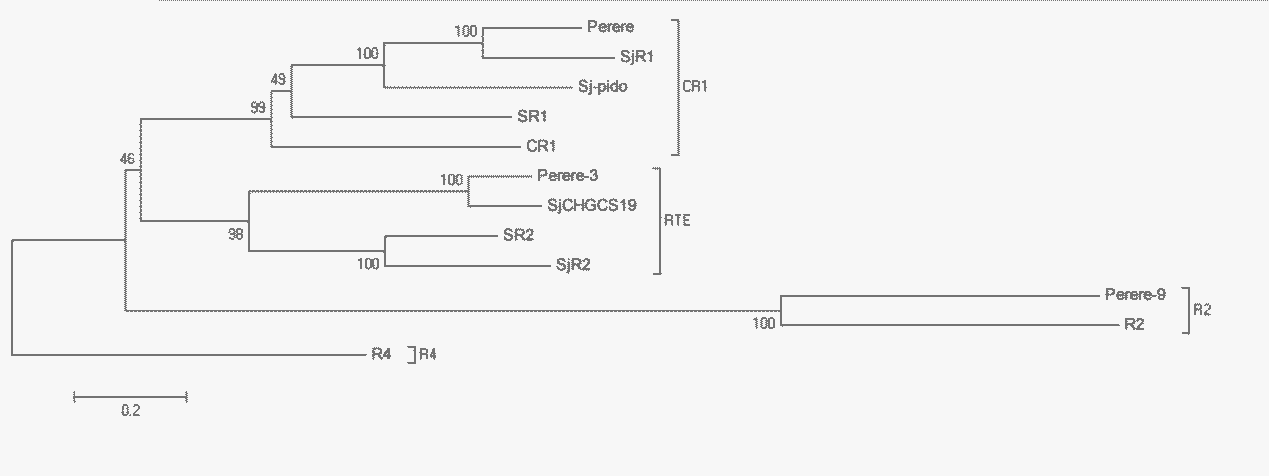

Supplement: Figure S2 — Phylogenetic relationshiops of the reverse transcriptase domain of SjCHGCS19 with other non-LTR retrontransposons. The tree was constructed by the Neighbor-Joining method. Numbers represent the percentage of replicate trees in which the associated taxa clustered together in the bootstrap test (1000 replicates). Rerere (DAA04497.1), Perere-3 (CAJ00236.1), Perere-9 (CAJ00246.1), SR1 (AAC06263.1) and SR2 (AAC24982.2) represent non-LTR retrotransposons of Schistosoma mansoni. SjR1 (AAC62955.1), Sj-pido (AY034003.1), SjR2 (AAK14815.1) and SjCHGCS19 (CAX83710.1) represent non-LTR retrotransposon of S. japonicum. CR1 (AAC60281.1) represents non-LTR retrotransposon of Gallus gallus, R2 (AAB59214.1) represents non-LTR retrotransposon of Bombyx mori, and R4 (AAA97394.1) represents non-LTR retrotransposon of Ascaris lumbricoides. (DOCX) [file pntd.0001579.s002.docx]
