# Supplementary material for: Sensitive and Specific Target Sequences Selected from Retrotransposons of Schistosoma japonicum for the Diagnosis of Schistosomiasis
Source: PLoS Negl Trop Dis. 2012 Mar 27;6(3):e1579. doi: 10.1371/journal.pntd.0001579 (PMC3313927; doi:10.1371/journal.pntd.0001579)
Supplement: Table S1 — The primers for the amplification of 26 Schistosoma japonicum retrotransposons by PCR. (DOC) [file pntd.0001579.s003.doc]

**Table S1.** The primers for the amplification of 26 *Schistosoma japonicum* retrotransposons by PCR

| **Retrotransposons** | **GenBank Accession No**. | **Primer 1 (forward)** | **Primer 2 (reverse)** | **Tm (℃)** |
| --- | --- | --- | --- | --- |
| ***LTR Retrotransposons*** | | | | |
| *SjCHGCS1* | FN356203.1 | GAATAATCCGCCCATCCA | AATTCTCGGCCTTTGATG | 55 |
| *SjCHGCS2* | FN356204.1 | CACCGTATGCCACCGAATC | CGAACGCTGTTTGGCACT | 53 |
| *SjCHGCS3* | FN356205.1 | CACAAGCCGCGATCCTAC | TGAGCCTCCTCCGTCCAT | 53 |
| *SjCHGCS4* | FN356206.1 | TAGGGCTGTTTGGCACGAA | GCAGCCGATGTGGGAGTT | --- |
| *SjCHGCS5* | FN356207.1 | TCTGCCCGTTCAAGAAGC | GCTCGTCTCGTGCCAACA | 53 |
| *SjCHGCS6* | FN356208.1 | AAGCACGGTGCCACTCCA | TCGGTGGTGGCATCGTCT | --- |
| *SjCHGCS7* | FN356209.1 | CGTTGATTATCTTGGCTTTG | AGACGGACCTGAACGACA | 55 |
| *SjCHGCS8* | FN3562010.1 | CTGCTTAGGTTTCGTGGAT | CCACAGGATCATCCCAAC | 55 |
| *SjCHGCS9* | FN3562011.1 | CAGCCACCTCGTAGGGTA | CACTCGCTGGTCCTCAAA | 55 |
| *SjCHGCS10* | FN3562012.1 | ATAGCCAGTGGGCATCTC | CTTTGCTGGAGCGAGTTT | 52 |
| *SjCHGCS11* | FN3562013.1 | GCCACCCGACAAACTCAA | ACGTAGCCGCTGAAAGGA | 54 |
| *SjCHGCS12* | FN3562014.1 | CCTCAATGAAGGCGACCCT | GCGTCCAATTCCAGGTGA | 54 |
| *SjCHGCS13* | FN3562015.1 | TTAGTGATGGACTGGGAGAA | CGTAGGCGATGAAGGTCA | 52 |
| *SjCHGCS14* | FN3562016.1 | ATGCTGCTCTGTCGGTTGT | AGGCGATGTAACTGTGGC | 52 |
| *SjCHGCS15* | FN3562017.1 | ACCAGTTTTCCGTCCAAA | TCCGTAGGTGCAGGCATT | 52 |
| *SjCHGCS16* | FN3562018.1 | ATGACCGAGGAGCTTTGA | TCGCATACAGAAGGCAAA | 52 |
| *SjCHGCS17* | FN3562019.1 | GGAGCGTTTCGATTGTTG | TGGTCCATGTCAGGTGCT | 52 |
| *SjCHGCS18* | FN3562020.1 | ACCCGATAGGGACGCTCTG | TAATGACCGACGCAAAGC | 52 |
| ***Non-LTR Retrotransposons*** | | | | |
| *SjR2* | AF412221 | TCTAATGCTATTGGTTTGAGT | TTCCTTATTTTCACAAGGTGA | 55 |
| *SjCHGCS19* | FN356221.1 | CCAAATCGCAACACTACGC | ATCGGATTCTCCTTGTTCAT | 55 |
| *SjCHGCS20* | FN356222.1 | ATCGTGAGCAGTGGTGGGC | GACAGTTCGCCATAAGCT | 53 |
| *SjCHGCS21* | FN356223.1 | TCTGCACCCGGTGTTGAT | GGAAACATTCAGGCGTTG | 54 |
| *SjCHGCS22* | FN356224.1 | CAAACACTCCTGAGGACCCA | GAGGCGTACATGGTGATCTAAC | 50 |
| ***Penelope-like elements*** | | | | |
| *Sj-penelope1* | FN356225.1 | CGCATCCATCGTTCCACT | TCCAGCCAGATACTTTGC | 50 |
| *Sj-penelope2* | FN356226.1 | CATCCCAATAGACACGCTTAA | CCGAGCACGGAGGAGTAA | 53 |
| *Sj-penelope3* | FN356227.1 | ACCCACAGGGACAACAAC | ACGGCACTGTAGAAATGAG | 50 |
